# Carotid sinus hypersensitivity and orthostatic hypotension in unexplained syncope

**DOI:** 10.1093/europace/euag295

**Published:** 2026-10-16

**Authors:** Sara Hassan, Hannes Holm, Frederik de Lange, Rose Anne Kenny, Michele Brignole, Artur Fedorowski

**Affiliations:** Department of Clinical Sciences, Lund University, Jan Waldenströms gata 35, Malmö 20502, Sweden; Department of Clinical Sciences, Lund University, Jan Waldenströms gata 35, Malmö 20502, Sweden; Department of Cardiology, Skåne University Hospital, Malmö 205 02, Sweden; Amsterdam University Medical Centre, University of Amsterdam, Heart Centre, Department of Clinical and Experimental Cardiology, Amsterdam Cardiovascular Sciences, Amsterdam, the Netherlands; Department of Medical Gerontology, Trinity College, Dublin, Ireland; Department of Cardiology, IRCCS Istituto Auxologico Italiano, Faint and Fall Research Centre, S. Luca Hospital, Piazzale Brescia 20, Milano 20149, Italy; Department of Clinical Sciences, Lund University, Jan Waldenströms gata 35, Malmö 20502, Sweden; Department of Cardiology, Karolinska University Hospital, and Department of Medicine, Karolinska Institute, Eugeniavägen 3, Stockholm (Solna) 171 76, Sweden

**Keywords:** Syncope, Orthostatic hypotension, Carotid sinus hypersensitivity, Carotid sinus massage, Head-up tilt, Cardiovascular autonomic testing

## Abstract

**Aims:**

Carotid sinus hypersensitivity (CSH) and orthostatic hypotension (OH) are established causes of syncope but are often considered separate entities. However, evidence suggests that CSH and OH may coexist and jointly contribute to syncope. This study investigates the relationship between CSH and OH in patients with unexplained syncope.

**Methods and results:**

A total of 1908 patients aged ≥40 years (age, 63.7 ± 13.0 years; men, 45.3%) with unexplained syncope underwent carotid sinus massage (CSM) and head-up tilt testing (HUT). Occurrence of CSH in classical and delayed OH, including asystolic CSM responses was analysed. Six-hundred-and-eighteen patients (32.4%, 68.9 ± 11.3 years) had OH, while CSH was present in 320 patients (16.8%, 72.2 ± 9.9 years), and was more common in patients with OH than without OH (24.1% vs. 13.3%, *P* < 0.001). Among patients with CSH, 46.6% also had OH. CSH occurred equally in both classical OH (62/275, 22.5%) and delayed OH (87/345, 25.2%). Among OH patients, CSH was associated with older age, male sex, lower supine diastolic blood pressure, hypertension, and syncope-related hospital admission. An asystolic CSM response was observed more often in OH patients (*n* = 42; 6.8%) compared with non-OH patients (*n* = 44, 3.4%, *P* < 0.001) and was associated with older age and lower resting heart rate.

**Conclusion:**

CSH and OH frequently coexist in adults over 40 years evaluated for syncope. CSM may reveal a clinically relevant cardioinhibitory reflex in a subgroup of OH patients, supporting comprehensive cardiovascular autonomic testing in unexplained syncope.

## Graphical Abstract

**Graphical Abstract:**
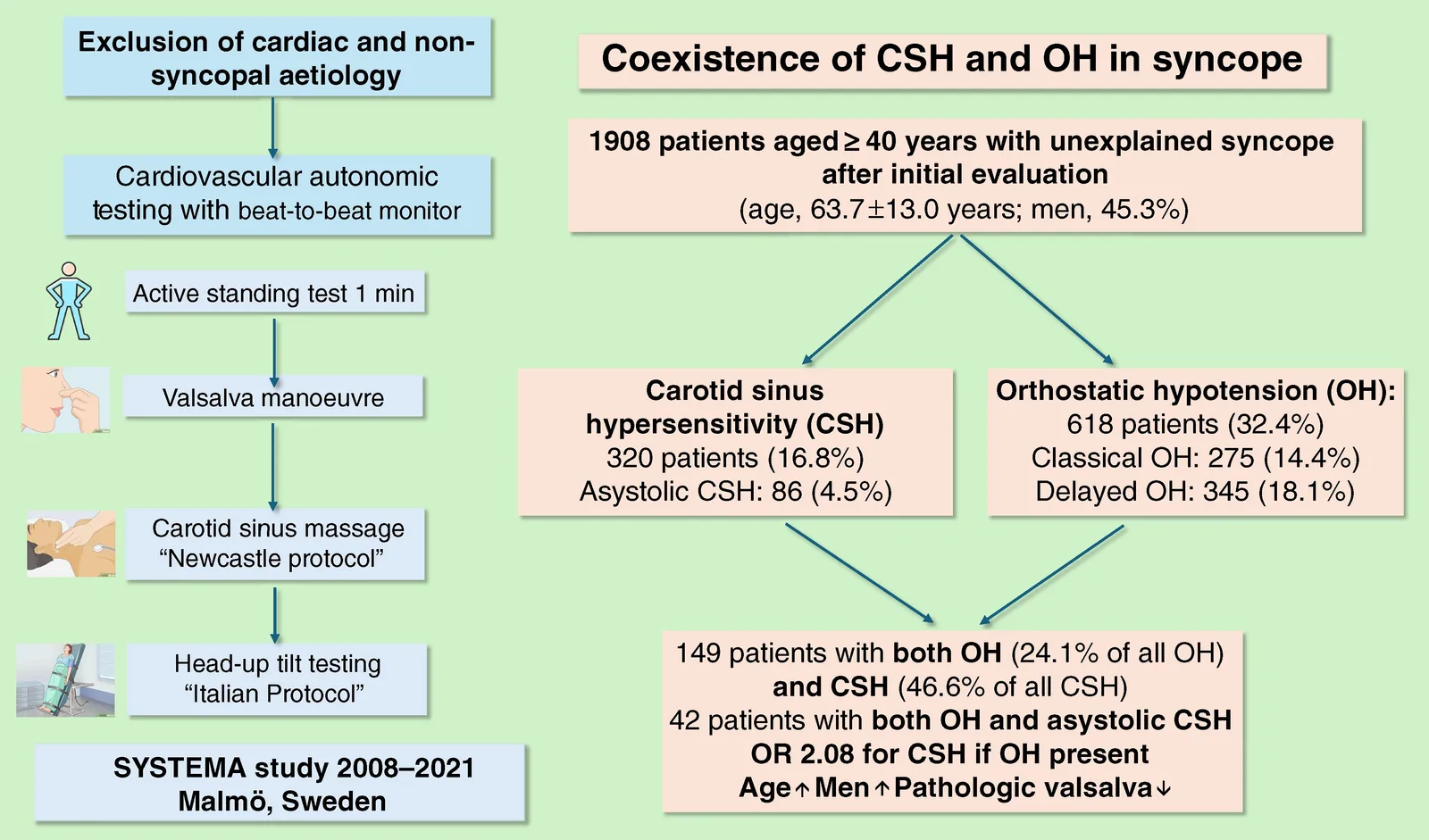

## Introduction

Although a substantial progress has been made over the past decades, syncope in older adults often remains difficult to diagnose.^1^ Episodes of syncope may be followed by amnesia for the event; previous studies reported that half to two-thirds of patients with syncope may not recall the LOC.^2^ As a result, syncope in elderly patients can be clinically missed or misinterpret as unexplained fall. A recent systematic review and meta-analysis showed that cardiovascular disorders, such as orthostatic hypotension (OH) and carotid sinus hypersensitivity (CSH), are associated with falls in older adults.^3^ This is important because among patients referred to a syncope unit for syncope evaluation, one in ten had suffered a fracture.^3^ In addition, older patients often present with two or more detectable conditions that could contribute to syncope, defined as complex syncope. These conditions may include reflex syncope, OH, CSH, cardiac arrhythmias and medication-related hypotension.^4–6^

The prevalence of OH increases with age from three percent to 35% in patients over the age of 75, as well as with comorbidities such as diabetes or Parkinson’s disease.^7,8^ Similarly to OH, CSH is more commonly observed in older individuals and may be clinically difficult to recognize because symptoms overlap with other causes of syncope.^5,9–11^ The cardioinhibitory form of CSH may be clinically relevant because it represents a potentially reversible cause of syncope through pacing or cardioneuroablation.^6,12–16^

Identifying the group of patients with OH, who also exhibit cardioinhibitory reflex may therefore have direct therapeutic implication.^13^ However, it is unclear whether the occurrence of carotid sinus hypersensitivity (CSH) is associated with presence of OH, and whether it differs between classic and delayed OH. Such information could contribute to a better understanding of the role of different autonomic pathways in syncope aetiology. Therefore, the aim with this study was to investigate whether CSH occurs in both classical and delayed forms of OH, and to evaluate the proportion of patients exhibiting a cardioinhibitory response.

## Material and methods

### Study design and population

This retrospective observational study was based on patients from previously described SYSTEMA cohort, which included patients referred to the tertiary syncope unit at Skåne University hospital in Malmö, Sweden for evaluation of unexplained syncope.^5^ Patients were recruited between 2008 and 2021. Referrals were reviewed by the principal investigator (AF) and colleagues, and, when deemed appropriate—i.e. when the mechanism of syncope remained unexplained after exclusion of cardiac causes and non-syncopal aetiologies based on the available medical records—patients were directly scheduled for cardiovascular autonomic testing. Patients aged ≥40 years who underwent both HUT and CSM were eligible for inclusion.

### Cardiovascular autonomic testing (CAT)

All patients underwent sequential cardiovascular autonomic testing using beat-to-beat haemodynamic monitor (Nexfin, BMEYE, Amsterdam, the Netherlands or Finapres NOVA, Finapres Medical Systems, Enschede, the Netherlands) including short one-min active standing test for transient immediate OH,^17^ seated Valsalva manoeuvre,^18^ CSM according to Newcastle protocol^19^ followed by HUT according to Italian protocol,^20^ consistent with the contemporaneous ESC syncope guidelines.^21^ CSM was performed bilaterally in the supine position and after one minute of HUT, followed by conventional HUT after 5-min rest. The 20-min passive HUT was used to assess orthostatic blood pressure response, including potential presence of classical and delayed OH. Occurrence of OH with symptom reproduction terminated the test. Nitroglycerine provocation was not applied in these patients. Additional clinical variables were obtained from the SYSTEMA database and from electronic medical records at the study site.

### Definitions and variables

Pathologic Valsalva response was defined as a progressive decline in BP in phase II, slow recovery of BP in phases III-IV, and absence of BP overshoot in phase IV.^22^ OH was defined as sustained decrease in systolic blood pressure (SBP) ≥ 20 mmHg and/or diastolic blood pressure (DBP) ≥ 10 mmHg during head-up tilt testing (HUT), including classical OH and delayed OH. Classical OH was defined as OH occurring within three minutes of HUT, while delayed OH was defined as OH occurring after 3 min of HUT.^6^ Carotid sinus hypersensitivity (CSH) was defined as a fall in systolic blood pressure ≥ 50 mmHg and/or ventricular pause ≥3 s during CSM.^6^ A cardioinhibitory CSH was defined as asystole ≥3 s during CSM.

The main variables analysed were CSM positivity, asystolic CSM response, classical and delayed OH. Clinical variables included age, sex, lifetime duration of syncope episodes i.e. time in years from first ever episode, the total number of syncope episodes, palpitations, supine syncope, traumatic falls, light-headedness on standing, supine SBP, supine DBP, supine heart rate, hypertension, diabetes mellitus, coronary artery disease, Parkinson’s disease, smoking status, and hospital admission due to syncope.

### Statistical analysis

Categorical variables were expressed as counts and percentages, while continuous variables were expressed as mean ± standard deviation (SD). Comparisons between groups were performed using Pearson’s chi-square tests for categorical variables and ANOVA for continuous variables. The primary outcome was the prevalence and co-occurrence of CSH and OH among patients undergoing cardiovascular autonomic testing. Secondary analyses characterized OH patients with and without CSH, and patients with an asystolic CSM response. Logistic regression models adjusted for age and sex were applied to identify predictors of CSM positivity in patients with OH. A two-sided *P*-value <0.05 was considered statistically significant. Statistical analyses were performed using IBM SPSS Statistics, version 31.0 (IBM Corp., Armonk, NY, USA).

### Ethical considerations

This study follows the declaration of Helsinki and is approved by the regional Swedish ethical review authority in Lund (dnr 82/2008). All participants gave written informed consent. Patient confidentiality was protected throughout the study, ensuring that patient integrity is not compromised. Access to medical records from Melior was restricted to study-related purposes only. No identifiable personal data were included in the analysis or presentation of results.

## Results

A total of 1908 patients aged 40 years or older (age, 63.7 ± 13.0 years; men, 45.3%) with unexplained syncope and valid data from both HUT and CSM were included in the main analysis. Proportions of CAT diagnoses among 1878 patients (missing values; *n* = 30) are presented in *Figure 1*. The most common diagnosis was vasovagal syncope (31.8%) followed by complex syncope^4^ i.e. presence of at least two contributing mechanisms: vasovagal reflex, OH, or CSH (21.8%). Forty-four patients (2.3%) had all three diagnoses i.e. vasovagal syncope, OH and CSH. There were no cases of tiOH-related syncope identified. Patient with negative CAT were followed up for recurrences or referred to further workup including use of implantable loop recorder, as appropriate. A total of 618 patients (32.4%) had OH: classical OH was present in 275 (14.4%) and delayed OH was present in 345 (18.1%) **(*Table 1*)**. In OH patients, the nadir SBP during HUT was 95.8 ± 21.6 mmHg and the maximal fall in SBP was 46.5 ± 23.7 mmHg, maximal fall in DBP was 14.3 ± 12.4 mmHg, and maximal HR increase was 12.5 ± 11.7 bpm. A total of 516 OH patients performed Valsalva test; of these 162 tests (31.4%) were pathological^18^ i.e. suggestive of cardiovascular autonomic failure and neurogenic cause of OH.^22^ Overall, CSH was identified in 320 patients (16.8%). CSH was more common among patients with any OH than among those without OH: 149 of 618 patients with OH (24.1%) had CSH, compared with 171 of 1290 patients without OH (13.3%; odds ratio 2.08 (1.63–2.66); *P* < 0.001 for intergroup difference) **(*Table 1*).** A total of 36 among 162 OH patients with pathologic Valsalva test also had positive CSM. Conversely, 46.6% of all patients with CSH also had OH. An asystolic CSM response was observed in 86 patients (4.5%), and the mean R–R interval was 7.0 ± 4.0 s; range 3.0–30.0 s. Asystolic CSM was observed in 6.8% patients with any OH (42/618), and 3.4% patients without OH (44/1290; odds ratio, 2.06 (1.34–3.19), *P* > 0.001 for intergroup difference).

**Figure 1:**
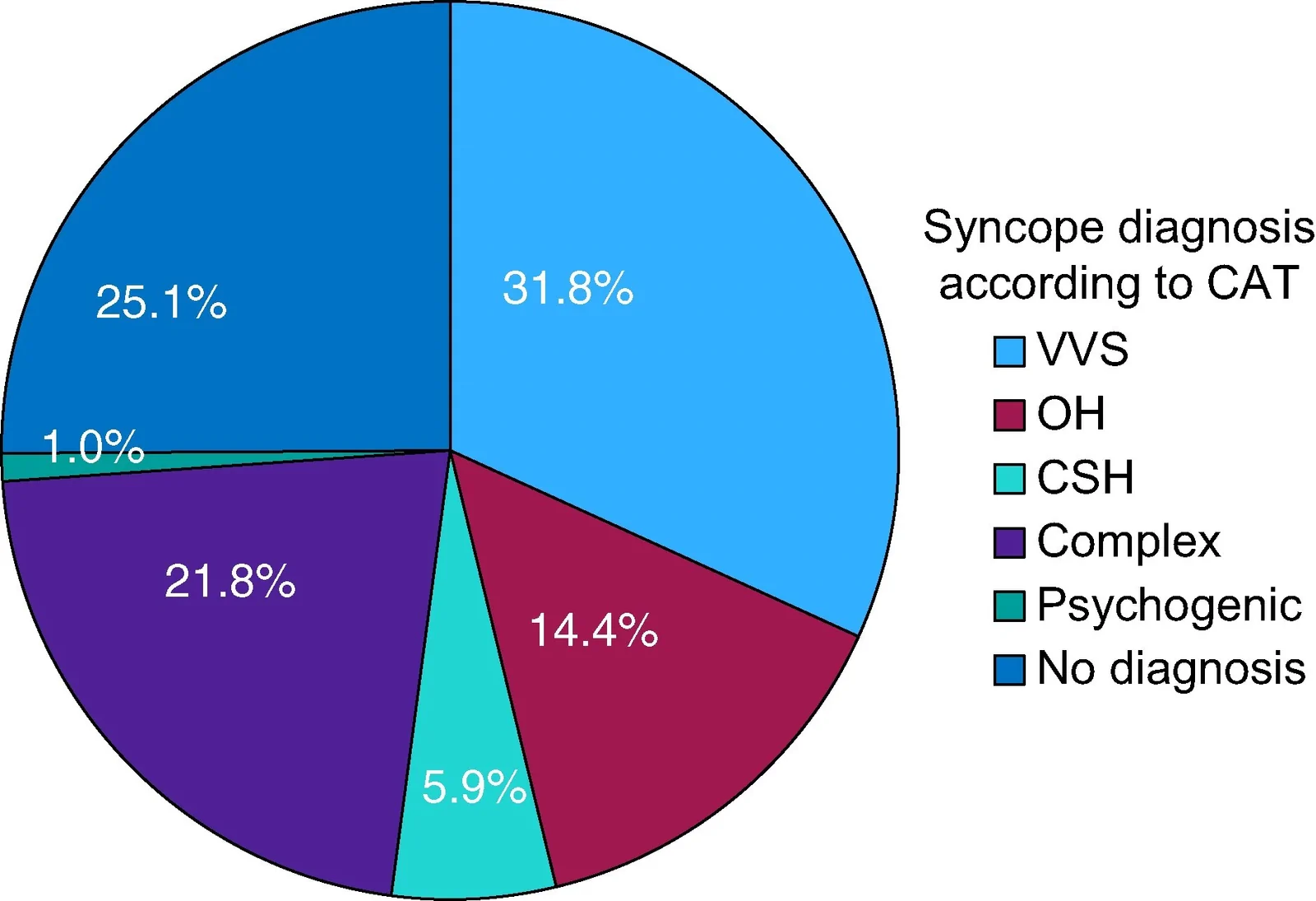
Proportions of syncope diagnoses obtained during cardiovascular autonomic testing including carotid sinus massage and tilt test. Adults aged ≥40 years (*n* = 1 878) with unexplained syncope after initial evaluation. Footnote: VVS, vasovagal syncope, *n* = 598 (31.8%); OH, orthostatic hypotension, *n* = 409 (14.4%); CSH; carotid sinus hypersensitivity, *n* = 110 (5.9%); Complex syncope i.e. presence of at least two: VVS, OH, or CSH, *n* = 409 (21.8%), psychogenic syncope mimic, *n* = 19 (1%); no diagnosis, *n* = 472 (25.1%).

**Table 1:** Prevalence of CSH and asystolic CSM response according to OH status

Variable	Total population	OH positive	OH negative	*P*-value
***n***	1908	618	1290	
**CSH positive, *n* (%)**	320 (16.8)	149 (24.1)	171 (13.3)	<0.001
**Asystolic CSM present, *n* (%)**	86 (4.5)	42 (6.8)	44 (3.4)	<0.001

CSH was equally present in both classical and delayed forms of OH. Among patients with classical OH, 62 of 275 (22.5%) had CSH, while 87 of 345 patients with delayed OH (25.2%) had CSH (*P* = 0.45). An asystolic CSM response was also observed in both OH subtypes: in 22 of 275 patients with classical OH (8.0%), and in 20 of 345 patients with delayed OH (5.8%, *P* = 0.34).

Among patients with OH, those with CSH were older compared with those without CSH (73.3 ± 8.9 vs. 67.5 ± 11.7 years, *P* < 0.001) and were more often men (62.4% vs. 50.5%, *P* = 0.011). They also had lower supine diastolic blood pressure (72.8 ± 11.4 vs. 76.6 ± 12.2 mmHg, *P* < 0.001). Hypertension was more common among OH patients with CSH (57.1% vs. 43.3%, *P* = 0.003), as was admission to the emergency department or hospital due to syncope (57.0% vs. 46.7%, *P* = 0.028). No statistically significant differences were observed for lifetime duration of syncopal episodes, number of syncopal episodes, supine SBP, supine heart rate, traumatic fall, light-headedness on standing, diabetes mellitus, Parkinson’s disease, or smoking status (***Table 2*)**. In the adjusted logistic regression model, positive CSM in OH patients was associated with advancing age [odds ratio (OR): 1.05 per one year, 95% confidence interval (CI) 1.03–1.07; *P* < 0.001], male sex (OR: 1.49, 95%CI 1.01–2.18; *P* = 0.046), normal Valsalva manoeuvre response (OR: 1.88, 95%CI, 1.14–2.94; *P* = 0.013) but not with SBP fall (*P* = 0.18), DBP fall (*P* = 0.17), or HR increase (*P* = 0.73) during HUT.

**Table 2:** Clinical characteristics among OH patients with and without CSH

Characteristic	CSH negative *n* = 469	CSH positive *n* = 149	*P*-value
Age, years	67.5 ± 11.7	73.3 ± 8.9	<0.001
Male sex, *n* (%)	237 (50.5%)	93 (62.4%)	0.011
Duration of symptoms (years)^a^	3 (1–10)	4 (1–10)	0.29
Number of syncope episodes^a^	4 (2–8)	3 (1–10)	0.12
Supine SBP, mmHg	142.6 ± 22.9	141.2 ± 22.5	0.503
Supine DBP, mmHg	76.6 ± 12.2	72.8 ± 11.4	<0.001
Supine heart rate, bpm	70.2 ± 11.3	68.8 ± 12.1	0.186
Hypertension, *n* (%)	202 (43.3)	84 (57.1)	0.003
Diabetes mellitus, *n* (%)	49 (10.4)	24 (16.2)	0.058
Traumatic fall, *n* (%)	255 (55.0)	76 (51.7)	0.490
Dizziness on standing, *n* (%)	345 (73.9)	101 (68.2)	0.181
Admitted to ED/hospital due to syncope, *n* (%)	218 (46.7)	85 (57.0)	0.028

Values are mean ± SD or *n* (%). Percentages are within CSH groups. ED, emergency department; SBP, systolic blood pressure; DBP, diastolic blood pressure.

^a^Presented as median (interquartile range)

An asystolic CSM response among the 42 patients with OH (mean duration, 6.7 ± 3.1 s; range, 3–19 s) was associated with older age (72.4 ± 9.0 vs. 68.7 ± 11.5 years, *P* = 0.042) and lower supine heart rate (64.9 ± 8.9 vs. 70.2 ± 11.6 beats per minute, *P* = 0.004) compared with the 107 patients with OH and non-asystolic CSH. Other clinical variables, including lifetime duration of syncopal episodes, total number of syncope episodes, supine SBP, and supine DBP, did not differ significantly between OH patients with and without an asystolic CSM response (***Table 3*)**. For asystolic CSM response, the results of logistic regression were similar to those for overall CSM positivity, but Valsalva test was no longer correlated with asystole during CSM (*P* = 0.18).

**Table 3:** Clinical characteristics of 149 OH patients with and without an asystolic CSH response

Characteristic	No asystolic CSM (*n* = 107)	Asystolic CSM (*n* = 42)	*P*-value
Age, years	68.7 ± 11.5	72.4 ± 9.0	0.042
Duration of symptoms (years)^a^	3 (1–10)	5 (1–16)	0.34
Number of syncope episodes^a^	4 (2–8)	3 (2–6)	0.11
Supine SBP, mmHg	142.2 ± 22.9	143.3 ± 21.0	0.770
Supine DBP, mmHg	75.9 ± 12.1	74.0 ± 12.0	0.340
Supine heart rate, bpm	70.2 ± 11.6	64.9 ± 8.9	0.004

Note. Values are mean ± SD. This table is based on the SPSS one-way analyses restricted to patients with OH.

^a^Presented as median (interquartile range)

## Discussion

The present study demonstrates that OH and CSH frequently coexist and may mutually or separately contribute to loss of consciousness in patients referred for evaluation of unexplained syncope. Nearly one-quarter of patients with OH also had CSH. Moreover, the asystolic response to CSM was more frequently encountered in syncope patients with OH than in those without OH. This challenges the traditional view that autonomic failure associated with OH is incompatible with carotid sinus-mediated abnormal cardiovascular responses.^23^

OH and CSH are both recognized causes of syncope, yet they are often thought of as distinct and unrelated aetiologically conditions. OH is typically attributed to impaired efferent autonomic regulation of blood pressure upon standing,^8,23^ whereas CSH is considered pathological exaggerated baroreceptor reflex, involving both afferent and efferent vagal and sympathetic pathways.^9,10,24^ The coexistence of CSH and OH highlights that syncope mechanisms in older patients are often multifactorial. In a study by Miller et al., the authors found hyperphosphorylated tau accumulation in baroreflex-associated nuclei, which may impair central regulation of baroreflex responses.^25^ Tau protein accumulation is a characteristic feature of neurodegenerative disorders such as dementia, movement disorders and motor neuron disease.^26^ In addition, the haemodynamic responses during HUT are unable to discriminate between different underlying OH aetiologies, as recently demonstrated.^27^

Current ESC syncope guidelines emphasize a structured initial evaluation through detailed history taking, physical examination and electrocardiogram (ECG).^6^ Guideline-recommended cardiovascular autonomic testing (CAT) includes CSM andHUT.^4,6^ Despite CSH and OH being both common in older population, there are sparse data on how often these occur simultaneously, particularly across classical and delayed forms of OH.^7^ This knowledge gap is clinically relevant because coexistence can affect both the diagnostic protocol for autonomic studies and subsequent choice of treatment.

Our results align with emerging evidence showing that CSH can occur alongside OH and other reflex mechanisms such as vasovagal syncope.^28–30^ The present study extends these findings by focusing specifically on the occurrence of CSH among patients with OH and demonstrating that OH should not necessarily be considered a sufficient explanation for syncope or unexplained falls in older adults.

In our study, 6.8% of patients with OH had an asystolic CSM response, and nearly half of all patients with an asystolic CSM response had OH. This suggests that syncope in these patients may not be explained by BP fall alone but may also involve reflex-mediated mechanisms that slow or briefly pause the heart. These findings support the use of both CSM and HUT in selected patients with unexplained syncope and falls, as recently proposed.^11^ This is particularly relevant in older adults, in whom syncope may present as an unexplained fall, especially when the event is unwitnessed or followed by amnesia.^2,29^ Bourke et al. showed in a systematic review and meta-analysis that OH was associated with increased odds of falls, and that cardiovascular disorders including OH and CSH could contribute to falls in older patients.^3^ Therefore, detecting one cardiovascular mechanism should not prevent further investigation for additional mechanisms. In particular, the observation of a significant CSM response should not terminate the diagnostic procedure; tilt testing should also be performed, as recently proposed in the 2STEPS trial.^11^ Conversely, identification of OH should not preclude CSM, particularly when the syncope scenario is unclear or has changed over time. Of note, abnormal response to CSM was also observed in patients with OH and pathologic Valsalva response, questioning the use of Valsalva test and diagnosis of neurogenic OH to determine who should undergo CSM.

OH and cardioinhibitory CSH may require different treatment strategies. OH is commonly managed thought medication review, volume expansion, compression garments, and measures to improve orthostatic tolerance, whereas cardioinhibitory carotid sinus syndrome may, in selected patients with symptom reproduction, be considered for pacemaker therapy.^6^ Failure to identify and assess both mechanisms may result in treatment being directed at only one component of the patient's syncope. Patients with coexisting OH and cardioinhibitory CSH may therefore require combined or sequential treatment strategies.

Clinical presentation did not clearly distinguish patients with both OH and CSH from those with OH alone. Lifetime duration of syncopal episodes, number of syncope episodes, traumatic falls and orthostatic intolerance symptoms were similar across groups. Patients with combined OH and CSH were older and more likely to be male, had lower supine diastolic BP, and more frequently had hypertension. The higher frequency of hypertension and tendency for lower supine diastolic BP may mirror the vascular ageing, altered baroreflex function or increased cardiovascular vulnerability.^2^ Lower baseline HR in patients with OH and asystolic CSH compared with those with non-asystolic CSH may suggest an associated intrinsic sinus node dysfunction that potentiates the vagal reflex.

These findings may be relevant for investigation of unexplained syncope after initial evaluation in older patients who commonly present with recurrent or unexplained symptoms and where the diagnostic uncertainty is greatest. In such settings, identifying coexisting mechanisms may improve diagnostic precision and treatment. Taken together, these results support the use of comprehensive cardiovascular autonomic testing in selected, particularly older patients with unexplained syncope.

## Strengths and limitations

A major strength of this study is the large sample size of patients aged 40 years or older who underwent both HUT and CSM in a specialized syncope unit. This made it possible to assess the coexistence of OH and CSH in a clinically relevant population. The use of standardized cardiovascular autonomic testing and the availability of detailed clinical variables further strengthen the descriptive characterization of the study population.

Several limitations should also be considered. First, the study was retrospective and observational, which limits the causal interpretation and therapeutic consequences of the results. Second, the cohort was derived from a single tertiary syncope unit, and the results may not be directly generalizable to other populations. Future prospective studies should evaluate whether systematic CSM in patients with unexplained syncope regardless of OH status improves diagnostic yield, treatment selection, and clinical outcomes.

## Conclusions

In this retrospective study of patients referred for syncope evaluation, CSH and OH frequently coexisted. CSH was present in approximately one-quarter of patients with OH and occurred in both classical and delayed OH. These findings support a comprehensive diagnostic approach in patients with unexplained syncope and suggest that CSH and OH should not be regarded as mutually exclusive conditions.

## Data Availability

The data underlying this article will be shared on reasonable request to the corresponding author.
